# Frequency‐dependent functional connectivity in resting state networks

**DOI:** 10.1002/hbm.25184

**Published:** 2020-08-25

**Authors:** Jessica Samogin, Marco Marino, Camillo Porcaro, Nicole Wenderoth, Patrick Dupont, Stephan P. Swinnen, Dante Mantini

**Affiliations:** ^1^ Research Center for Motor Control and Neuroplasticity KU Leuven Leuven Belgium; ^2^ Brain Imaging and Neural Dynamics Research Group IRCCS San Camillo Hospital Venice Italy; ^3^ Institute of Cognitive Sciences and Technologies (ISTC) National Research Council (CNR) Rome Italy; ^4^ Centre for Human Brain Health and School of Psychology University of Birmingham Birmingham UK; ^5^ Department of Information Engineering Università Politecnica delle Marche Ancona Italy; ^6^ Research in Advanced Neurorehabilitation S. Anna Istitute Crotone Italy; ^7^ Neural Control of Movement Lab, Department of Health Sciences ETH Zurich Zurich Switzerland; ^8^ KU Leuven Brain Institute KU Leuven Leuven Belgium; ^9^ Laboratory for Cognitive Neurology KU Leuven Leuven Belgium

**Keywords:** functional connectivity, high‐density electroencephalography, neuronal communication, resting state, time‐frequency analysis

## Abstract

Functional magnetic resonance imaging studies have documented the resting human brain to be functionally organized in multiple large‐scale networks, called resting‐state networks (RSNs). Other brain imaging techniques, such as electroencephalography (EEG) and magnetoencephalography (MEG), have been used for investigating the electrophysiological basis of RSNs. To date, it is largely unclear how neural oscillations measured with EEG and MEG are related to functional connectivity in the resting state. In addition, it remains to be elucidated whether and how the observed neural oscillations are related to the spatial distribution of the network nodes over the cortex. To address these questions, we examined frequency‐dependent functional connectivity between the main nodes of several RSNs, spanning large part of the cortex. We estimated connectivity using band‐limited power correlations from high‐density EEG data collected in healthy participants. We observed that functional interactions within RSNs are characterized by a specific combination of neuronal oscillations in the alpha (8–13 Hz), beta (13–30 Hz), and gamma (30–80 Hz) bands, which highly depend on the position of the network nodes. This finding may contribute to a better understanding of the mechanisms through which neural oscillations support functional connectivity in the brain.

## Graphical abstract

Functional magnetic resonance imaging studies have documented the resting human brain to be functionally organized in multiple large‐scale networks, called resting‐state networks (RSNs). We examined frequency‐dependent functional connectivity between the main nodes of several RSNs, spanning large part of the cortex. We observed that functional interactions within RSNs are characterized by a specific combination of neuronal oscillations in the alpha (8–13 Hz), beta (13–30 Hz), and gamma (30–80 Hz) bands, which highly depend on the position of the network nodes.

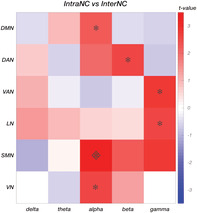

## 1 INTRODUCTION

Functional magnetic resonance imaging (fMRI) studies documented that the resting human brain is functionally organized in several large‐scale networks, so‐called resting‐state brain networks (RSNs) (Damoiseaux et al., 2006; Fox & Raichle, 2007; Raichle et al., 2001). These RSNs were named in analogy to topologically corresponding brain networks that are modulated during task performance. For instance, RSNs such as the default‐mode (DMN), the dorsal (DAN) and the ventral (VAN) attention, the language (LN), the somatomotor (SMN) and the visual (VN) networks are typically reported in resting‐state fMRI studies (Damoiseaux et al., 2006; Smitha et al., 2017). Considering that fMRI provides only an indirect measure of neural activity, other brain imaging techniques such as electroencephalography (EEG) and magnetoencephalography (MEG) were also proposed to study RSNs (Brookes et al., 2014; de Pasquale et al., 2010; Liu, Farahibozorg, Porcaro, Wenderoth, & Mantini, 2017; Marino et al., 2018; Samogin, Liu, Marino, Wenderoth, & Mantini, 2019; Siems, Pape, Hipp, & Siegel, 2016; Tang et al., 2017; Yuan et al., 2016). Although EEG and MEG have lower spatial resolution compared to fMRI, they are better suited to investigate the electrophysiological correlates of functional connectivity in RSNs. Indeed, they provide a direct measure of neural activity and their temporal resolution is sufficiently high to capture fast neural oscillations in the brain (de Pasquale et al., 2010; Mantini, Perrucci, Gratta, Romani, & Corbetta, 2007; Marino, Arcara, Porcaro, & Mantini, 2019). Neural activity measured using EEG/MEG is classified based on the frequency of its oscillations, in the delta (1–4 Hz), theta (4–8 Hz), alpha (8–13 Hz), beta (13–30 Hz), and gamma (30–80 Hz) bands (Atasoy, Deco, Kringelbach, & Pearson, 2018; Buzsáki, 2006; Roopun et al., 2008). The role of neural oscillations in supporting brain network connectivity is yet unclear. By using simultaneous EEG‐fMRI in participants at rest, we revealed that fMRI activity in brain networks were correlated with power fluctuations of neuronal oscillations, primarily in the alpha, beta and gamma bands (Mantini et al., 2007). More recent MEG and high‐density EEG (hdEEG) studies (Samogin et al., 2019; Tang et al., 2017), which focused on the DMN, confirmed that the alpha band oscillations play a pivotal role in supporting functional interactions between all network nodes, and documented that beta and gamma band oscillations support interactions between relatively closer node pairs (Samogin et al., 2019). It remains to be elucidated whether and to which extent the findings obtained for the DMN generalize to other RSNs. Several studies suggested that oscillations at higher and lower frequencies may support short‐ and long‐range connectivity patterns, respectively (Jones, Pinto, Kaper, & Kopell, 2000; Kopell, Ermentrout, Whittington, & Traub, 2000; Lopes da Silva, 2013). Further elaborating on this concept, we posited that the neural oscillations supporting functional connectivity between network nodes may relate to their spatial distribution over the cortex (Ganzetti & Mantini, 2013). In the present study, we investigated frequency‐dependent connectivity within several RSNs using hdEEG, thereby extending the work we recently conducted for the DMN (Samogin et al., 2019). We tested the hypothesis that connectivity in the alpha band is the most prominent in the resting state condition not only for the DMN but also for other RSNs. A second hypothesis that was tested, however, was that other frequency bands may show the largest difference between within‐network and between‐network connectivity.

## 2 MATERIALS AND METHODS

### 2.1 EEG data collection

EEG data were collected in 19 healthy young adult volunteers (age 28 ± 6 years, 14 females) during an eyes‐open resting state condition. They were previously used in one of our previous studies (Samogin et al., 2019). Ethical approval was granted by the Ethics Committee of ETH Zurich. The experiment was performed in accordance with the relevant guidelines and regulations, and informed consent was obtained from all participants. For each participant, we recorded hdEEG signals for 5 min at 1000 Hz sampling rate using a 256‐channel HydroCel Geodesic Sensor Net by Electrical Geodesics (Eugene, OR). Vertical electrooculogram (vEOG), horizontal electrooculogram (hEOG) and electromyogram (EMG) were collected in addition to the EEG signals. Positions of the EEG sensors as well as of three landmarks (nasion, left and right preauricular) were localized using a Geodesic Photogrammetry System (Russell, Eriksen, Poolman, Luu, & Tucker, 2005). Moreover, we acquired in a separate session a T1‐weighted whole‐head anatomical image using a Philips Ingenia 3T Magnetic Resonance (MR) scanner (Best, The Nederlands) with a turbo field echo sequence. The scanning parameters were: TR = 8.25 ms, TE = 3.8 ms, flip angle = 8°, voxel size = 1 mm^3^ isotropic.

### 2.2 fMRI data collection

Eyes‐open resting state fMRI data were collected in a different cohort of 24 healthy volunteers (25.5 ± 5.5 years, 15 females). These data have been already used in previous studies (Liu, Ganzetti, Wenderoth, & Mantini, 2018; Mantini et al., 2012; Mantini & Vanduffel, 2013; Samogin et al., 2019). Ethical approval was granted by the Ethics Committee of Chieti University. The experiment was performed in accordance with the relevant guidelines and regulations, and informed consent was obtained from all participants. Functional images were obtained using a Philips Achieva 3T MR scanner. More specifically, T2*‐weighted echo‐planar imaging (EPI) with blood oxygen level dependent (BOLD) contrast was used. The scanning parameters were the following: 32 axial slices, 230 × 230 in‐plane matrix, TR = 2000 ms, TE = 35 ms, flip angle = 90°, voxel size = 2.875 × 2.875 × 3.5 mm^3^, 300 dynamic volumes. Furthermore, we acquired a 3D high‐resolution T1‐weighted whole‐head anatomical image using an MP‐RAGE sequence, used as anatomical reference. The scanning parameters were: TR = 9.1 ms, TE = 3.7 ms, flip angle = 8°, voxel size = 0.938 × 0.938 × 1 mm^3^.

### 2.3 EEG data analysis

We used an automated analysis workflow for studying frequency‐dependent functional connectivity from EEG data. This workflow was used in a previous study (Samogin et al., 2019), and consisted of four main steps: EEG signals preprocessing, individual head model creation, reconstruction of EEG source space data, seed‐based connectivity analysis.

#### 2.3.1 EEG signals preprocessing

The first step was the cleaning of the EEG data, to correct bad channels and to attenuate noise and biological artifacts (Liu et al., 2017; Samogin et al., 2019). First of all, we detected channels with low signal quality and label them as “bad channels.” To this end, we used an automated procedure that combines information from two different parameters. The first parameter was the minimum Pearson correlation of the signal in the band (1–80 Hz) against all the signals from the other channels. The second parameter was the noise variance in the band 200–250 Hz, where the contribution of the EEG signal can be considered negligible. We defined bad channels those channels for which at least one of the two channel‐specific parameters was an outlier as compared to the total distribution of values. To ensure robustness of the detection, the threshold to define an outlier was set to *m* + 4*s*, where *m* was the average value and s was the standard deviation. Subsequently, the signal of each bad channel was reconstructed by spatially interpolating the neighboring channels, as defined using the FieldTrip toolbox (http://www.fieldtriptoolbox.org/). Next, we band‐pass filtered the resulting EEG data in the band (1–80 Hz) using EEGLab (https://sccn.ucsd.edu/eeglab), and we re‐referenced them in average reference, by removing the mean value across channels (Liu et al., 2015). We attenuated ocular and muscular artifacts that were present in the EEG recordings by using independent component analysis (ICA) (D. Mantini, Franciotti, Romani, & Pizzella, 2008). Specifically, we used a fast fixed‐point ICA (FastICA) algorithm (http://research.ics.aalto.fi/ica/fastica) with deflation approach and hyperbolic tangent as contrast function (Hyvarinen, 1999) to estimate independent components (ICs), as well as the weights with which those ICs were mixed in the data. The ICs associated with the artifacts (or artifactual ICs) were automatically identified using the artifact detection solution implemented in (Liu et al., 2017). This relies on the following parameters: 1) correlation of the power of the IC with the power of vEOG, hEOG and EMG signals; 2) the coefficient of determination obtained by fitting the IC power spectrum with a 1/f function; 3) the kurtosis of the IC time‐course. An IC was classified as artifactual if at least one of those parameters was above its specific threshold, set in accordance with previous studies (de Pasquale et al., 2010; Liu et al., 2017; Dante Mantini, Mantini, Corbetta, Perrucci, Romani, & Del Gratta, 2009). The artifact‐corrected EEG signals were obtained by linearly mixing the ICs that were not classified as artifactual, with the corresponding weights estimated by ICA.

#### 2.3.2 Individual head model creation

A realistic head model was reconstructed from the anatomical MR image, which was previously segmented in 12 different compartments, and the EEG sensors positions, which were rigidly co‐registered to the head contour (Liu et al., 2017; Samogin et al., 2019). On the layers corresponding to cortical, subcortical, and cerebellar gray matter, a 3D regular 6 mm grid was overlapped in order to define all the possible dipole sources. Conductivity values were chosen based on previous literature (Haueisen, Ramon, Eiselt, Brauer, & Nowak, 1997). Finally, the whole‐head finite element head model was generated by using SimBio (Wolters, Grasedyck, Anwander, & Hackbusch, 2004; Ziegler et al., 2014). Based on this head model, a leadfield matrix expressing the linear relationship between scalp EEG data and source‐space neural activity was calculated.

#### 2.3.3 Reconstruction of EEG source space data

The exact low‐resolution brain electromagnetic tomography (eLORETA) algorithm was used to compute the cortical three‐dimensional distribution of current density from processed scalp EEG data (Pascual‐Marqui et al., 2011). The algorithm, which also used the leadfield matrix as input, estimated source‐space neural activity in a 6 mm homogeneous grid constrained to the gray matter.

#### 2.3.4 Seed‐based connectivity analysis

**FIGURE 1:**
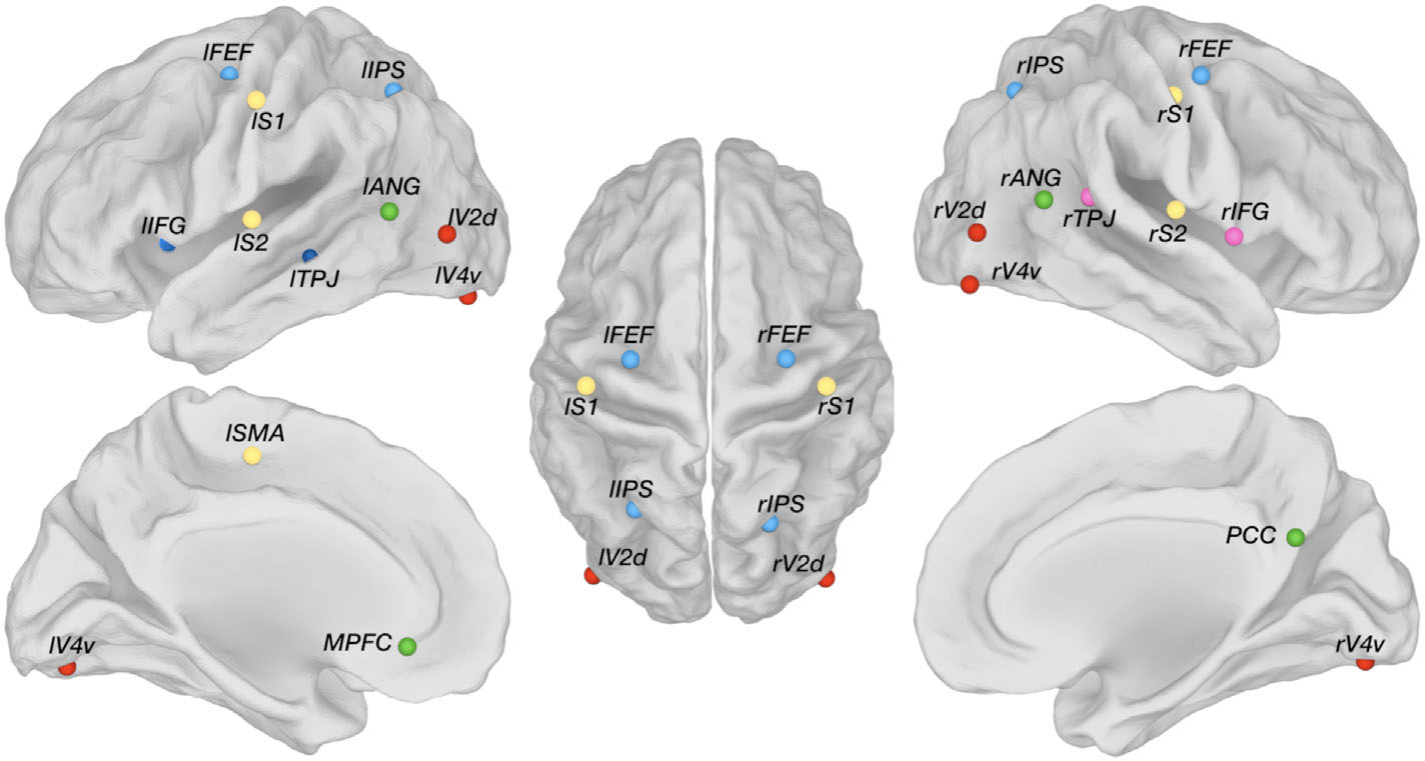
Anatomical positions of the 21 seeds used in the study, subdivided into the corresponding networks: *Default mode network* (DMN, green); *Dorsal attention network* (DAN, light blue); *Ventral attention network* (VAN, pink); *Language network* (LN, dark blue); *Somatomotor network* (SMN, yellow); *Visual network* (VN, red). MNI coordinates can be found in Table S1, whereas the full names are listed here: Posterior cingulate cortex (PCC), Medial prefrontal cortex (MPFC), Left angular gyrus (lANG), Right angular gyrus (rANG); Left Frontal Eye Field (lFEF), Right Frontal Eye Field (rFEF), Left Inferior Parietal Sulcus (lIPS), Right Inferior Parietal Sulcus (rIPS); Right Temporo‐Parietal Junction (rTPJ), Right Inferior Frontal Gyrus (rIFG); Left Temporo‐Parietal Junction (lTPJ), Left Inferior Frontal Gyrus (lIFG); Left Supplementary Motor Area (lSMA), Left Primary Somatosensory Cortex (lS1), Right Primary Somatosensory Cortex (rS1), Left Secondary Somatosensory Cortex (lS2), Right Secondary Somatosensory Cortex (rS2); Left human ventral Visual 4 area (lV4v), Right human ventral Visual 4 area (rV4v), Left dorsal Visual 2 area (lV2d), Right dorsal Visual 2 area (rV2d)

We examined connectivity between the nodes of six RSNs, which have been most commonly investigated in previous studies, and cover together large part of the cortical surface: DMN, DAN, SMN, VN, VAN, and LN. Among these RSNs, the last two are strongly lateralized, whereas the others are bilateral. The main nodes of the RSNs were selected based on previous studies (de Pasquale et al., 2012; Grootswagers, Cichy, & Carlson, 2018; D. Mantini et al., 2007; Samogin et al., 2019), imposing a minimum distance of 15 mm between them to minimize the spurious effects of signal leakage. Accordingly, 21 nodes were defined in the Montreal National Institute (MNI) space (Table S1 and Figure 1). The node coordinates were projected to individual space. For each of them, a spherical region of interest (ROI) with 6 mm radius was defined. Time‐courses corresponding to voxels in the gray‐matter were decomposed in the time‐frequency domain using the short‐time Fourier transform. We calculated the power spectrum of each ROI in the range (1–80 Hz), and then reconstructed the power spectrum of each RSN by averaging those constituting its ROIs. From the RSN power spectra, we extracted the power in the delta (1–4 Hz), theta (4–8 Hz), alpha (8–13 Hz), beta (13–30 Hz), and gamma (30–80 Hz) bands. For each of these bands, we tested whether the power of pairs of RSNs was different, by using a paired two‐tailed *t*‐test. The false discovery rate (FDR) method (Benjamini & Hochberg, 1995) was used to account for multiple comparisons across bands and RSNs, its significance level was set to *q <* 0.001. Next, EEG connectivity was measured using power envelope correlations between orthogonalized signals (Hipp, Hawellek, Corbetta, Siegel, & Engel, 2012). Pearson correlations were calculated on the logarithmic‐transformed signal‐orthogonalized power time‐courses. These correlations were then transformed to z‐values using the Fisher transform (de Pasquale et al., 2012; Hipp et al., 2012). Connectivity maps corresponding to the delta, theta, alpha, beta, and gamma brain oscillations were reconstructed by averaging the z‐values calculated for each individual frequency within the relevant range. Connectivity maps were registered to MNI space to assess the statistical significance of the connectivity results across participants. Specifically, we performed a one‐sample *t* test for each frequency band on the EEG connectivity maps in MNI space. The FDR value *q* was calculated to account for multiple comparisons and its significance level was set to *q <* 0.05. Then, we examined the EEG connectivity profiles between pairs of RSNs, for each frequency band. In particular, we defined intra‐network connectivity (IntraNC) as the average connectivity between pairs of ROIs within a specific network. Similarly, inter‐network connectivity (InterNC) was calculated as the average connectivity between all the possible pairs of ROIs belonging to two different networks (Newton, Morgan, Rogers, & Gore, 2011). Frequency‐specific IntraNC and InterNC values, respectively, were averaged within the five frequency bands of interest (delta, theta, alpha, beta, and gamma). For each frequency band and for each pair of RSNs, we then regressed out the difference in power from the connectivity values. In order to test for the effects of frequency band and network on IntraNC (and InterNC) values, we used a two‐way analysis of variance (ANOVA). Furthermore, a two‐tailed paired *t*‐test was performed on IntraNC and InterNC values, for each frequency band and each RSN. The FDR method was used to account for multiple comparisons, and the significance level was set to *q *< 0.05.

### 2.4 fMRI data analysis

FMRI data were preprocessed using standard procedures for functional connectivity analyses, including: head motion correction, registration to brain anatomy, band‐pass filtering (0.01–0.1 Hz), regression of head motion (3 translation and 3 rotation parameters), white matter, cerebrospinal fluid and global signals, and spatial smoothing at 6 mm full width half maximum (de la Cruz et al., 2019). Seed coordinates were projected in individual MR space and around each coordinate a spherical ROI was defined, with 6 mm radius. FMRI connectivity maps were calculated by correlating the fMRI time‐course of the seed ROI with the time‐courses of all the voxels in the gray matter. fMRI connectivity maps of each individual were then registered to MNI space, and a group‐level connectivity map was obtained by calculating a one‐sample t‐test across them. The FDR value *q* was calculated to account for multiple comparisons, and its significance level was set to *q < 0.05*.

### 2.5 Comparison of EEG and fMRI connectivity maps

Spatial matching of EEG and fMRI connectivity maps calculated using the same seed was achieved quantitatively using the dice similarity index (DSI) (Dice, 1945). The EEG and fMRI connectivity maps were binarized using the significance level *q <* 0.05 as threshold, such that the spatial overlap could be quantified using DSI. The statistical significance of DSI values was assessed using a Monte Carlo approach with 300 iterations. For each iteration, surrogate EEG source signals with similar frequency content were generated using the iterative amplitude adjusted Fourier transform (IAAFM) method (Schreiber & Schmitz, 1996). With this new time‐course, a pseudo‐EEG connectivity map was produced using the same approach used for real source‐localized EEG activity. Subsequently, the DSI between pseudo‐EEG and fMRI connectivity maps was calculated and logged. This process yielded a total of 300 DSI values, which were used as “null‐distribution” to calculate a probability associated with the true DSI between EEG and fMRI connectivity maps.

## 3 RESULTS

**FIGURE 2:**
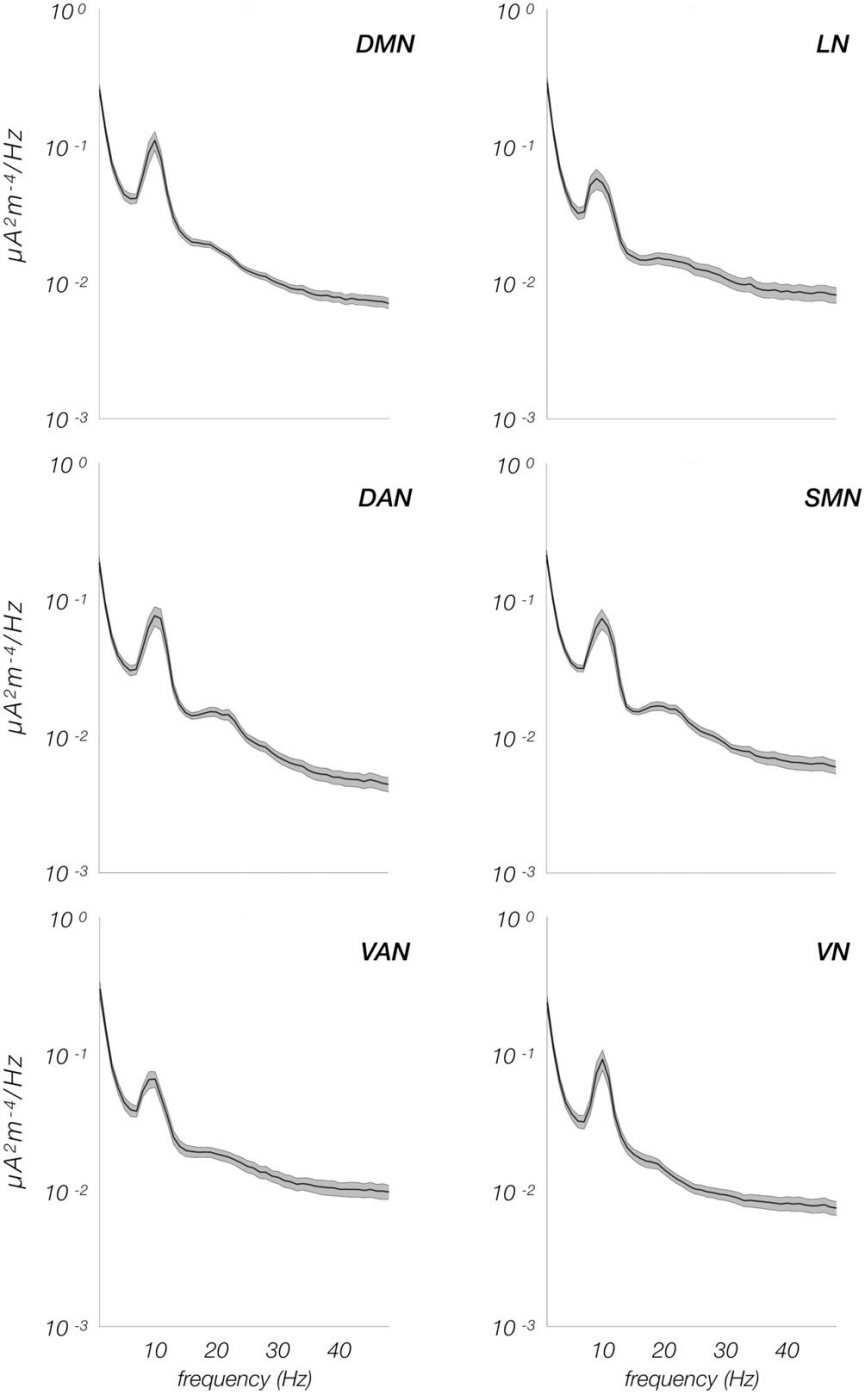
Power spectra of the six resting state networks. In each panel, the black line represents the average power across the participants, whereas the surrounding gray shaded area corresponds to the standard error of the mean

**FIGURE 3:**
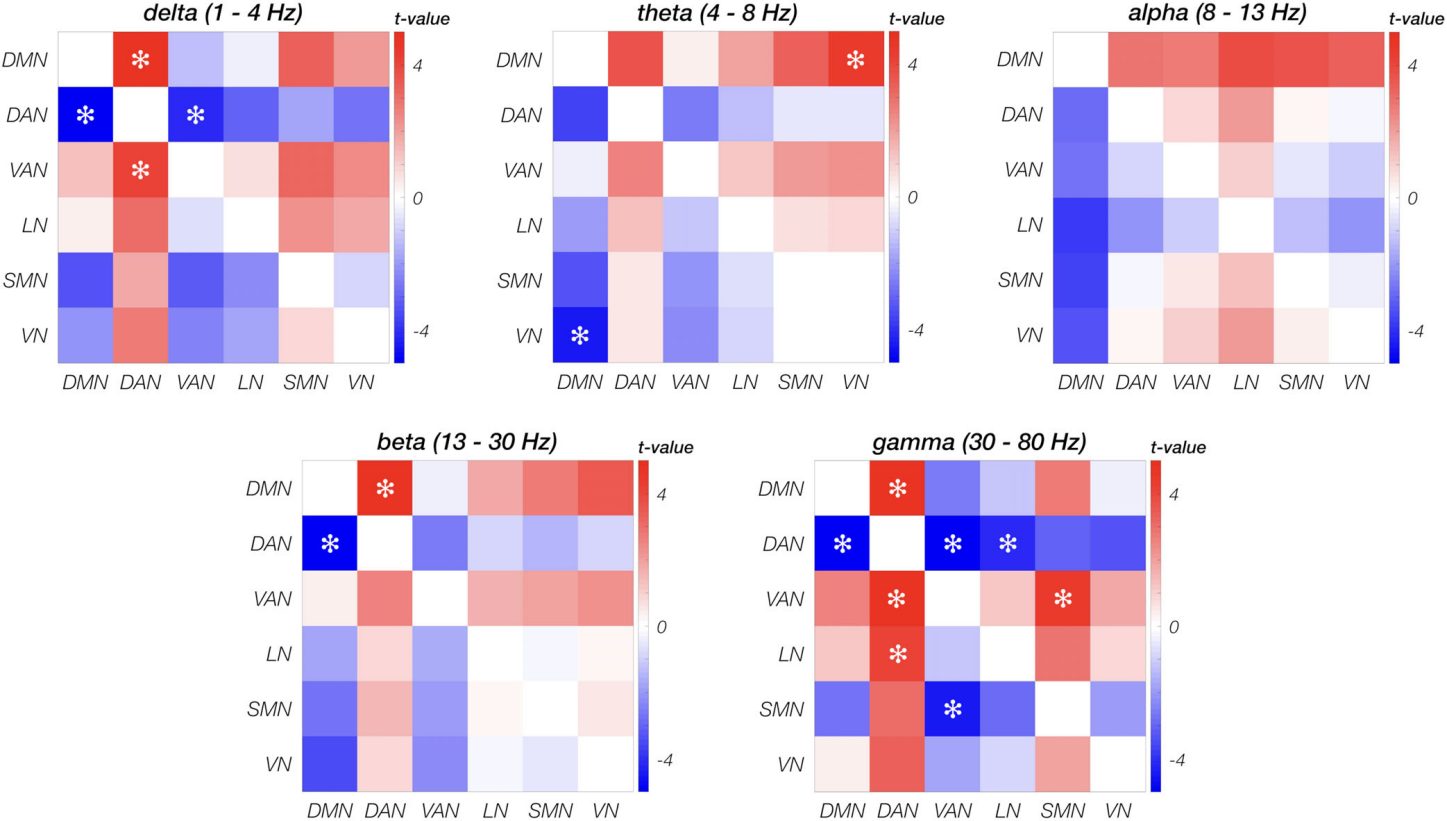
Comparison between power spectra of the RNSs. For all network pairs, a paired two‐tailed *t* test was used on the power values averaged over the frequencies within each band (delta, theta, alpha, beta, gamma). Differences that are significant at *p* < .001 are marked with an asterisk, whereas those at *q <* 0.001 with a diamond

The six RSNs under investigation had relatively similar power spectral density profile, characterized by stronger values in the delta band, a prominent peak in the alpha band and a smaller one in the beta band (Figure 2). Despite this overall similarity in power spectral density profile, the RSN power was found to be significantly different in specific frequency bands (Figure 3). In particular, the DMN had significantly larger power than DAN in delta (*p* = .0001), beta (*p* < .0001), and gamma (*p* < .0001) bands, and than VN in the theta band (*p* = .0003). In the gamma band, the power of DAN was significantly lower than that of VAN (*p* = .0001) and LN (*p* = .0006), respectively, and the power of VAN was significantly larger than that of SMN (*p* = .0003). Finally, DAN and VAN had significantly different power not only in the gamma, but also in the delta band (*p* = .0006).

**FIGURE 4:**
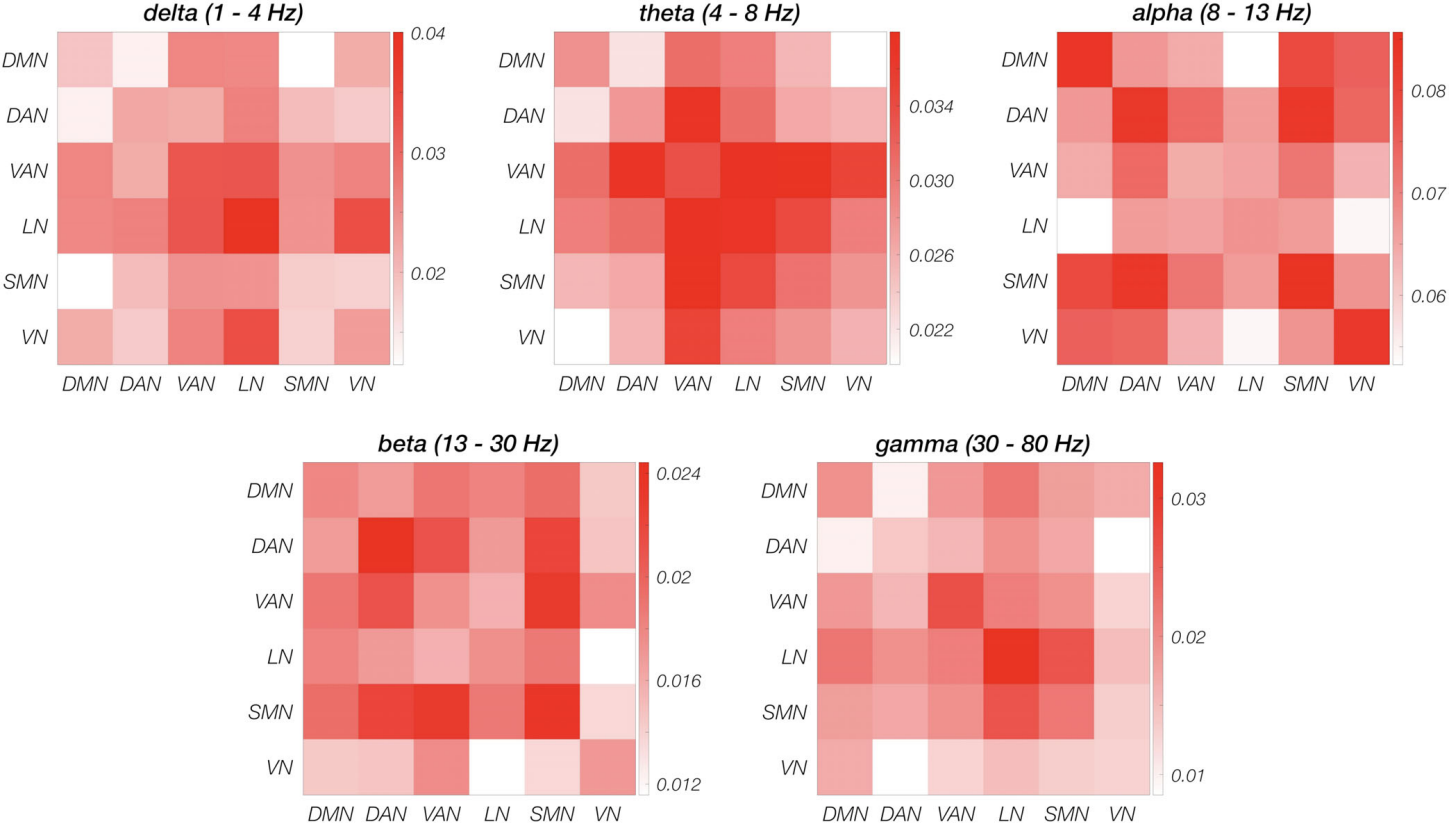
Functional connectivity values between pairs of networks in the five frequency bands (delta, theta, alpha, beta and gamma). In each panel, group average connectivity values on the diagonal are calculated from the intra‐network connectivity (IntraNC) values, whereas the upper (lower) triangular matrix correspond to the inter‐network connectivity (InterNC) measures, both averaged over the subjects

When analyzing network connectivity profiles (Figure 4 and Figure S1), we found that they differed according to the frequency band considered. For each network, IntraNC was higher than InterNC for specific frequency bands. For example, DMN, SMN, and VN showed higher values in the alpha band for IntraNC than for InterNC. Conversely, IntraNC values peaked for VAN and LN in the gamma band and for DAN in the beta band. Within the same frequency band, the InterNC values largely varied, highlighting different coupling strengths between pairs of networks.

**FIGURE 5:**
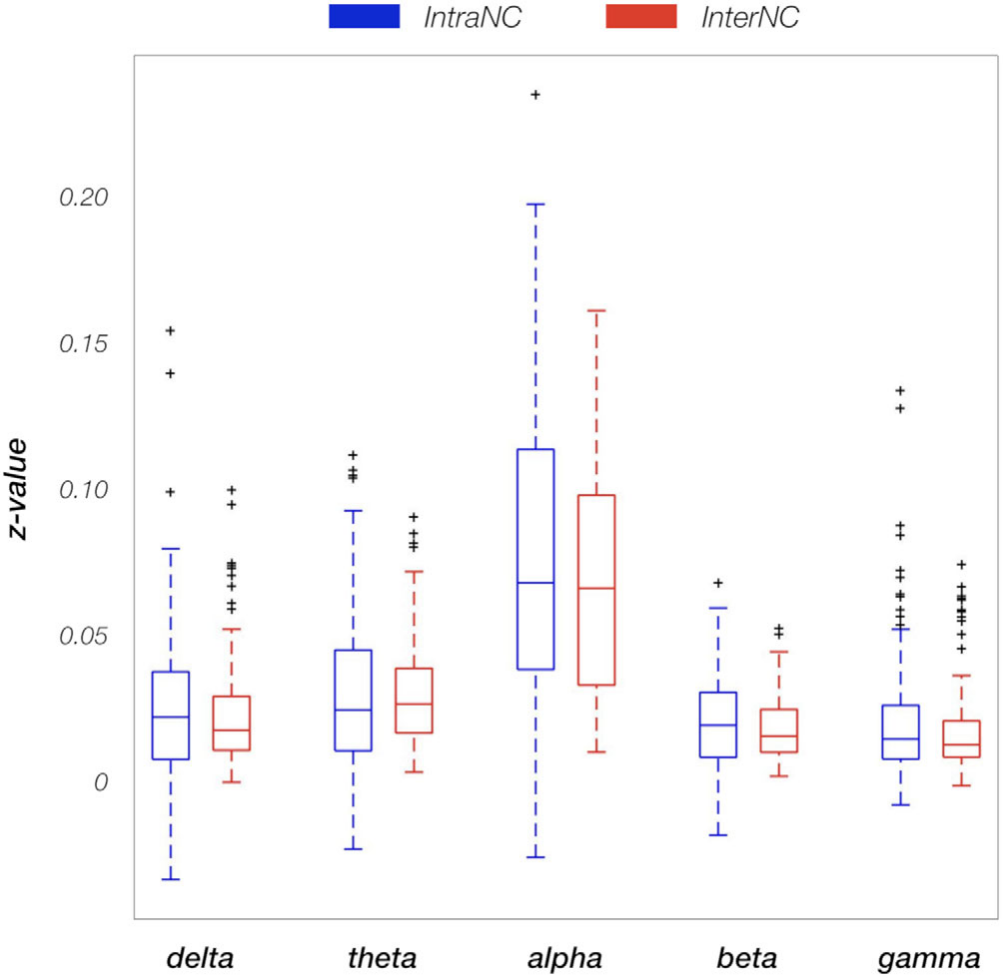
Box plot of all the intra‐network connectivity (IntraNC, blue) and inter‐network connectivity (InterNC, red) values for the 19 participants, calculated in each frequency band. Correlation values are Fisher‐transformed. Outliers outside the interquartile range are plotted as black crosses

**FIGURE 6:**
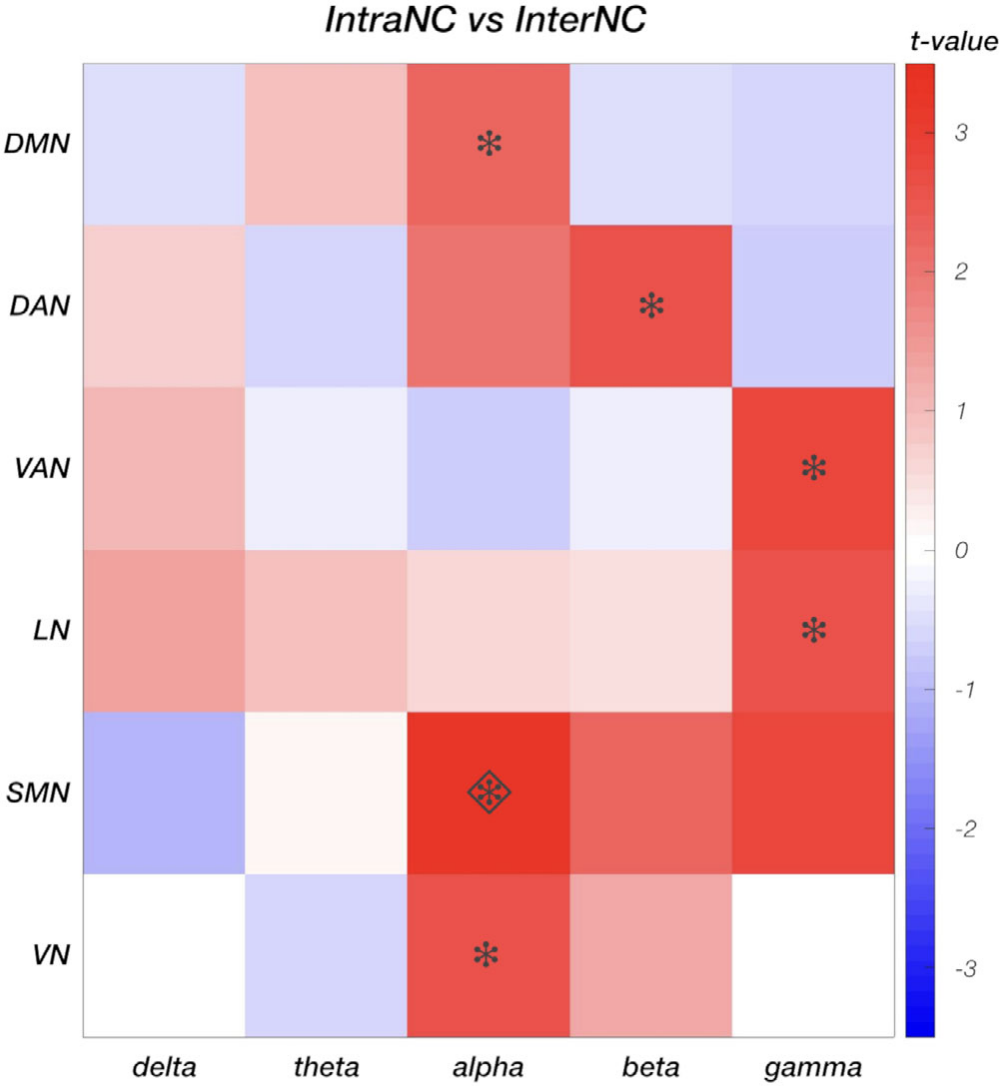
Comparison between intra‐ and inter‐ network connectivity (IntraNC and InterNC, respectively), for each pair of networks in the five frequency bands (delta, theta, alpha, beta and gamma). A two‐tailed paired t‐test was used to compare IntraNC and InterNC values. Differences that are significant at *p <* .05 are marked with an asterisk, whereas those at *q <* 0.05 with a diamond

ANOVA tests on IntraNC and InterNC values (Figure 5 and Figure S2), revealed large variability across frequency bands (*p* < .001, for both IntraNC and InterNC), but not RSNs (*p* = .794 and *p* = .799 for IntraNC and InterNC, respectively). Post hoc paired *t* tests between pairs of frequency bands showed that both IntraNC and InterNC values were larger (*q* < 0.001) in the alpha band than in any other band (Figure S3). Moreover, IntraNC and InterNC values were larger (*q* < 0.001) in the theta band as compared with the beta and gamma bands, respectively. InterNC in the delta band was lower from that in theta and gamma bands (both *q* < 0.001). In addition, IntraNC was significantly larger than InterNC in the alpha, the beta, and the gamma frequency bands for all the networks (Figure 6). In particular, the strongest differences between IntraNC and InterNC values was reached in the alpha band for DMN (*p* = .0242, *q* = 0.0908), SMN (*p* = .0011, *q* = 0.0335) and VN (*p* = .0100, *q* = 0.0562), in the beta band for DAN (*p* = .0112, q = 0.0562) and in the gamma band for VAN (*p* = .0055, q = 0.0549) and LN (*p* = .0089, q = 0.0562).

**FIGURE 7:**
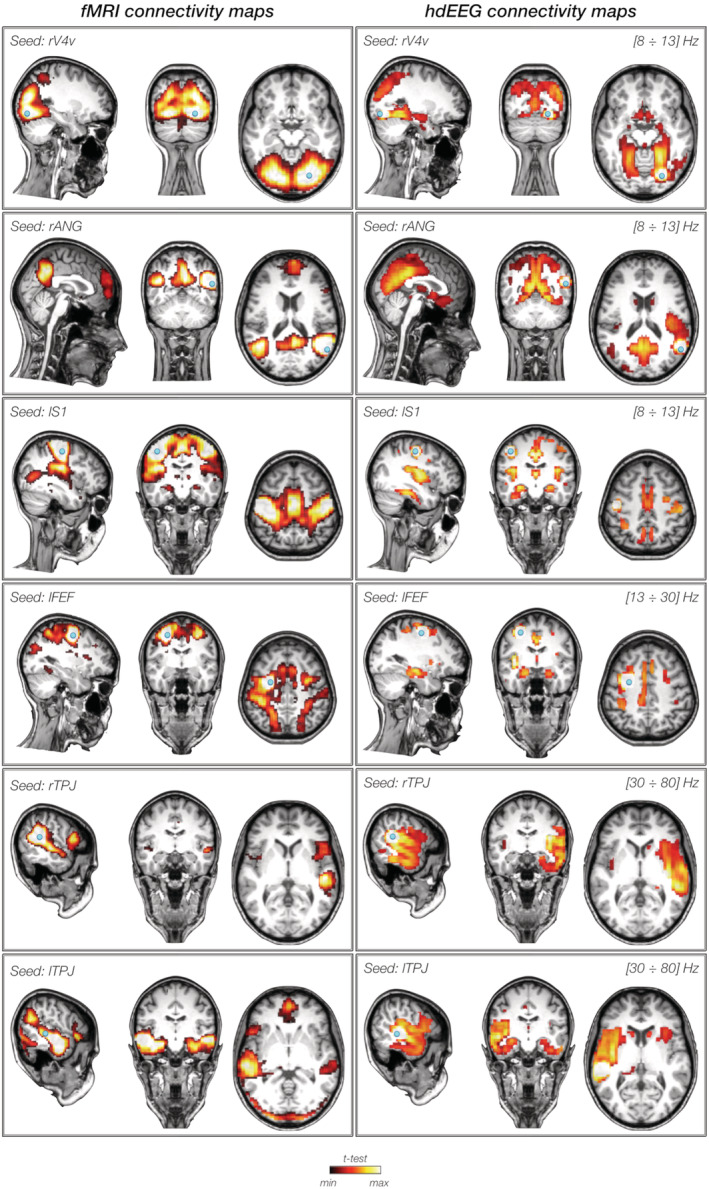
Seed based connectivity maps from fMRI data (left) and hdEEG data (right). The presented maps are associated with six seeds, one for each network (from top panel): rV4v in the visual network, rANG in the default mode network, lS1 in the somatomotor network, lFEF in the dorsal attention network, rTPJ in the ventral attention network and lTPJ in the language network. EEG connectivity was calculated in the bands for which IntraNC was found significantly higher than InterNC: alpha band (8–13 Hz) for rV4v, rANG, and lS1, beta band (13–30 Hz) for lFEF and gamma (30–80 Hz) for rTPJ and lTPJ. Group level spatial maps are shown in coronal, sagittal, and axial sections, thresholded at *q <* 0.05

Notably, the EEG connectivity maps obtained for individual seeds, reconstructed in the frequency bands for which the difference between IntraNC and InterNC was the strongest, qualitatively and quantitatively matched the fMRI connectivity map calculated using the same seed (Figure 7 and Figure S4). Connectivity was relatively lower in the EEG as compared to the fMRI maps, particularly for brain regions around the seed. However, the EEG maps showed the seed region to be connected to topologically‐distant brain regions, typically coherent with those emerging from the fMRI analysis.

## 4 DISCUSSION

The results of our study indicated that hdEEG can be reliably used to map RSNs in the human brain, and the source localization of hdEEG signals provides sufficient spatial resolution to disentangle brain regions involved in different networks. We were therefore able to use this technique to investigate which neural oscillations support connectivity in RSNs. Notably, hdEEG connectivity was generally most prominent in the alpha band, but the largest similarity with fMRI connectivity was obtained for some networks when oscillations in the beta and gamma frequency bands were considered. In general, the neural oscillations for which the difference between IntraNC and InterNC values was the strongest seemed to depend on the specific network topology. We will elaborate on the points above in the following paragraphs.

### 4.1 Imaging of RSNs using hdEEG

In this study, we measured functional connectivity of source‐reconstructed hdEEG data, between a set of nodes of interest distributed within six RSNs (Figure 1). To this end, we used an analysis workflow for hdEEG preprocessing, source localization and seed‐based connectivity analysis, which was previously validated using a single RSN, specifically the DMN (Samogin et al., 2019). The reconstructed connectivity maps for all RSNs showed great variability depending on the frequency band in which they were calculated. Notably, some of those connectivity maps were remarkably similar with the corresponding spatial maps obtained from the fMRI data set (Figure 7). This finding confirmed that seed‐based connectivity analysis could be performed using hdEEG, as an alternative to the commonly used MEG (M. J. Brookes et al., 2011; O'Neill, Barratt, Hunt, Tewarie, & Brookes, 2015) and fMRI techniques (Biswal, Zerrin Yetkin, Haughton, & Hyde, 1995; Lee, Smyser, & Shimony, 2013; van den Heuvel & Hulshoff Pol, 2010). The limited costs and the portability of hdEEG systems may open important avenues for a more widespread use of this technique, not only to address basic neuroscientific questions but also for applied or clinical research.

### 4.2 Role of alpha oscillations in network connectivity

Regardless of the network, we found higher IntraNC and InterNC values in the alpha band compared to all the other frequency bands (Figure 5 and Figure S3). This may be due to the fact that alpha is the rhythm dominating brain activity at rest (de Munck et al., 2007; Marino et al., 2019; Roopun et al., 2008; Tyvaert, LeVan, Grova, Dubeau, & Gotman, 2008). It may indeed be argued that there is a common generator that activates neural assemblies spread all over the cortex and increases the true connectivity between them. A structure that is extensively connected with the cortex (Behrens et al., 2003; Behrens, Berg, Jbabdi, Rushworth, & Woolrich, 2007) and is known to strongly contribute to the generation of alpha oscillations is the thalamus (de Munck et al., 2007; Hughes & Crunelli, 2005; Tyvaert et al., 2008). This brain region has been recognized to be a promoter of global communication and information integration across the brain (Malekmohammadi, Elias, & Pouratian, 2015; Wang, Leong, Chan, Liu, & Wu, 2019). Indeed, brain oscillations in the alpha band spread through thalamocortical connections to stimulate local activity (X. Wang et al., 2019). Similarly to the cortical functional organization, which is preserved even when the brain is not actively responding to a specific stimulus, such thalamocortical pathways could be present and detectable also during resting state. To address this research question, future studies would need to be conducted to examine thalamocortical and corticocortical connectivity in the alpha band, as well as in other frequency bands.

### 4.3 Relationship between network topology and neural oscillations

In addition to the high connectivity values in the alpha band (Figure 5 and Figure S3), we detected significant differences in connectivity strength between nodes of the same and different networks, respectively (Figure 6). Notably, empirical results from our study suggest that differences in IntraNC and InterNC values across RSNs are not directly related to differences in their activity levels (Figure 3). The frequency bands for which IntraNC values were significantly larger than InterNC ones were not only the alpha but also the beta and the gamma bands, depending on the RSN considered. For some RSNs, as for instance the DMN, the DAN, the VN, and the SMN, we found neural oscillations supporting network connectivity that are largely in line with previous EEG‐fMRI (D. Mantini et al., 2007; Marino et al., 2019), MEG (de Pasquale et al., 2010, 2012) and hdEEG studies (Samogin et al., 2019). Whereas the aforementioned networks were associated with the alpha and beta oscillations, the VAN and LN were primarily related to gamma oscillations. To the best of our knowledge, little experimental evidence exists in support to the association between RSNs and neural activity in the gamma band (but see Mantini et al., 2007). Accordingly, we suggest that future studies should be conducted to replicate our findings. Considering that VAN and LN are strongly lateralized as compared to DMN, DAN, VN, and SMN, our findings corroborate our hypothesis that the preferential neural oscillation for network connectivity depends on the spatial distribution of the RSN nodes. Previous studies suggested that slower oscillations are better suited than faster oscillations for supporting long‐range connectivity in the brain (Jones et al., 2000; Kopell et al., 2000; Lopes da Silva, 2013). Recent studies are addressing the question of how fMRI and MEG connectivity depends on the specific pattern of structural connections in the brain (Deco et al., 2014; Fukushima et al., 2018; Surampudi et al., 2018). In this regard, future computational modeling work focused on hdEEG connectivity may provide novel insights into the mechanisms through which neural oscillations support connectivity in brain networks, and in particular in generating new hypotheses on the possible relationships between network topology and preferential (alpha/beta/gamma) oscillations.

### 4.4 Study limitations

A number of limitations of this study should be mentioned. First we performed source localization using the eLORETA algorithm (Pascual‐Marqui et al., 2011), in line with our previous studies (Liu et al., 2017, 2018; Samogin et al., 2019; Zhao, Marino, Samogin, Swinnen, & Mantini, 2019). It has however been shown that each source localization method has different effects on EEG connectivity estimates (Anzolin et al., 2019). Furthermore, we measured functional connectivity using power envelope correlations between orthogonalized signals (Hipp, Hawellek, Corbetta, Siegel, & Engel, 2012). Several other connectivity methods are however available, each of them capturing slightly different features of the neural signals they were applied to. We therefore suggest that future studies should be conducted to test whether the main findings in this study can be replicated using different source localization algorithms and connectivity methods. Finally, we would like to point out that 21 ROIs associated with the main nodes of six different RSNs were included in the present study. This allowed us to test our hypotheses concerning intra‐ and inter‐network EEG connectivity. Nonetheless, to further investigate the relation between frequency‐specific connectivity and the spatial distribution of the network nodes, additional ROIs belonging to different RSNs should be considered. When the number of ROIs increases up to the point they are very close to each other, the use of pruning methods for connectivity analysis, such as hyperedge bundling (S. H. Wang et al., 2018), is warranted.

## 5 CONCLUSION AND FUTURE PERSPECTIVES

We have shed new light on the neural oscillations that primarily support intrinsic interactions within specific large‐scale networks. We have found evidence supporting the hypothesis that position of the nodes of a given RSN over the cortex influences the frequency of the neural oscillations related to network connectivity (Ganzetti & Mantini, 2013). This finding may represent an important step toward a better understanding of the mechanisms through which neural oscillations support functional connectivity in the brain. In future studies, it would be interesting to investigate how frequency‐dependent connectivity changes across different levels of consciousness (Cavanna, Vilas, Palmucci, & Tagliazucchi, 2018; Pal et al., 2019) and is modulated by task performance (Watrous, Tandon, Connor, Pieters, & Ekstrom, 2013). Moreover, future research may focus on characterizing changes in frequency‐dependent network connectivity during aging (King et al., 2017) as well as in neurological disorders (Bourgeron, 2009; Uhlhaas & Singer, 2006, 2010).

## CONFLICT OF INTEREST

The author declares that there is no conflict of interest.

## Supporting information


**Fig. S1.** Functional connectivity values between all possible pairs of seeds in five frequency bands (delta, theta, alpha, beta and gamma). Connectivity values are averaged across participants.

**Fig. S2.** Boxplot of intra‐network connectivity (IntraNC, blue) and inter‐network connectivity values (InterNC, red) for each network separately: connectivity values are Fisher‐transformed. *Default Mode Network* (DMN), *Dorsal Attention Network* (DAN), *Ventral Attention Network* (VAN), *Language Network* (LN), *Somatomotor Network* (SMN), *Visual Network* (VN).

**Fig. S3.** Differences in intra‐network (IntraNC) and inter‐network (InterNC) connectivity values between frequency bands. A two‐tailed paired t‐test was used to compare (A) IntraNC and (B) InterNC values, respectively, for each pair of frequency bands. Differences that are significant at *p<0.001* are marked with an asterisk, and those at *q<0.001* with a diamond.

**Fig. S4.** Dice similarity indices (DSI) between the EEG‐based and the fMRI‐based connectivity maps shown in Fig. 7 (clockwise from top left): rv4V (VN), rANG (DMN) and lS1 (SMN) in alpha band, rTPJ (VAN) and lTPJ (LAN) in gamma band, lFEF (DAN) in beta band. The actual DSI value are indicated in each panel with a dashed vertical line. A null‐distribution of DSI values, represented using a gray‐shaded histogram, was obtained by comparing the fMRI‐connectivity map with surrogate EEG‐connectivity maps.

**Table S1** **–** For each network, the selected seeds and their coordinates in MNI space are indicated.

## Data Availability

The authors do not have permission to share raw data. The data that support the findings of this study are available from the corresponding author upon reasonable request.
