# Supplementary figures and images for: Frequency‐dependent functional connectivity in resting state networks
Source: Hum Brain Mapp. 2020 Aug 25;41(18):5187–98. doi: 10.1002/hbm.25184 (PMC7670639; doi:10.1002/hbm.25184)

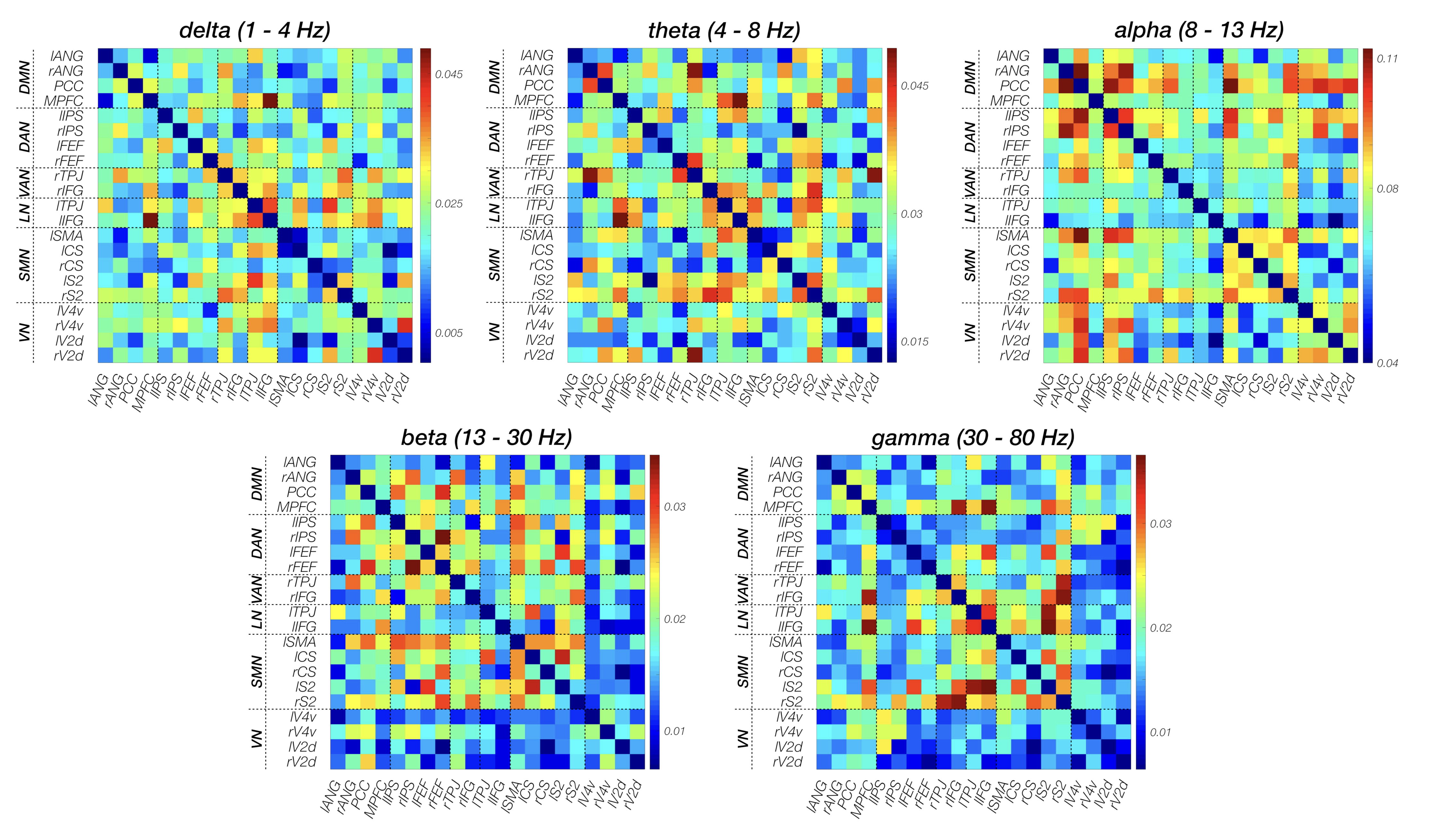

Supplement: Supplementary file 1 — Fig. S1. Functional connectivity values between all possible pairs of seeds in five frequency bands (delta, theta, alpha, beta and gamma). Connectivity values are averaged across participants. [file HBM-41-5187-s001.tiff]

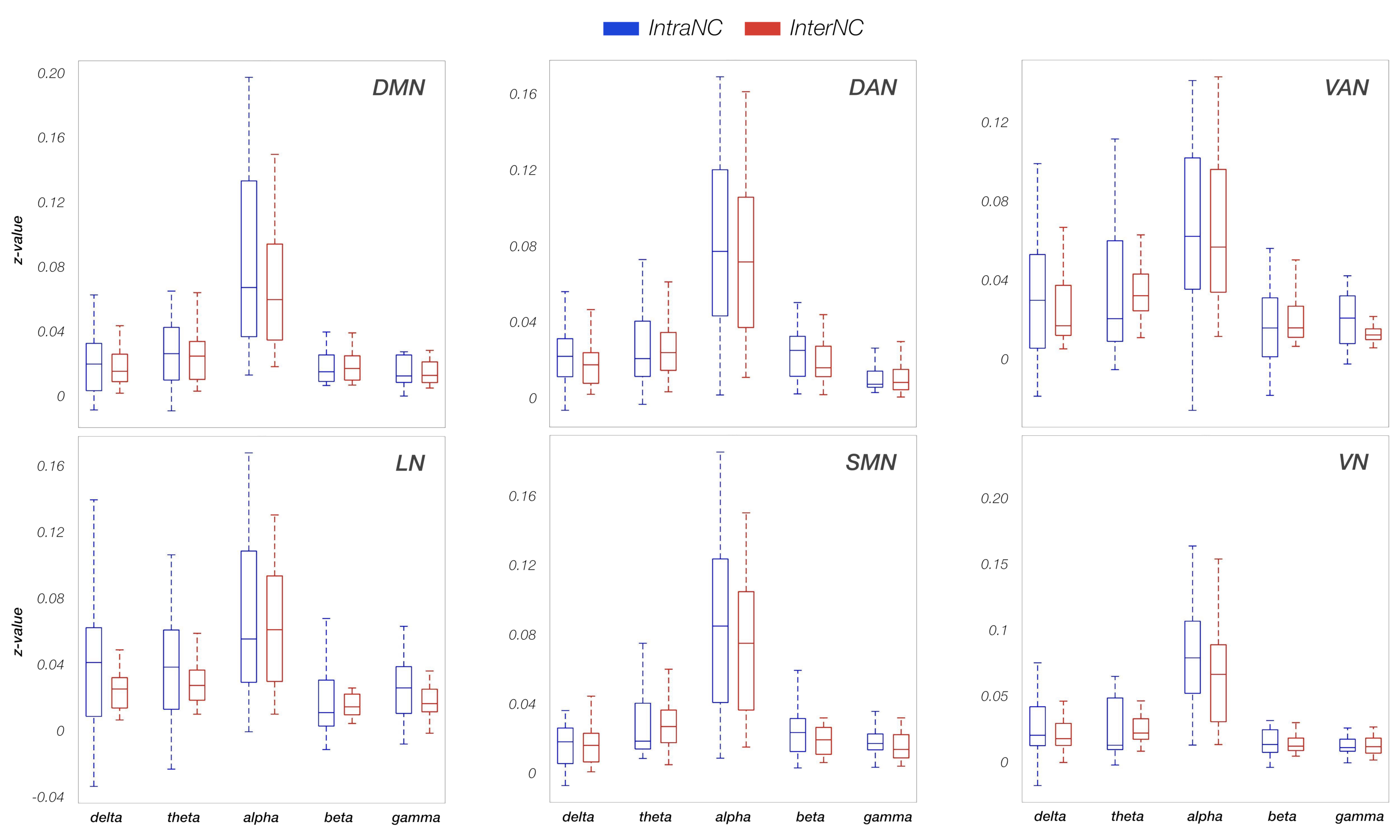

Supplement: Supplementary file 2 — Fig. S2. Boxplot of intra‐network connectivity (IntraNC, blue) and inter‐network connectivity values (InterNC, red) for each network separately: connectivity values are Fisher‐transformed. Default Mode Network (DMN), Dorsal Attention Network (DAN), Ventral Attention Network (VAN), Language Network (LN), Somatomotor Network (SMN), Visual Network (VN). [file HBM-41-5187-s002.tiff]

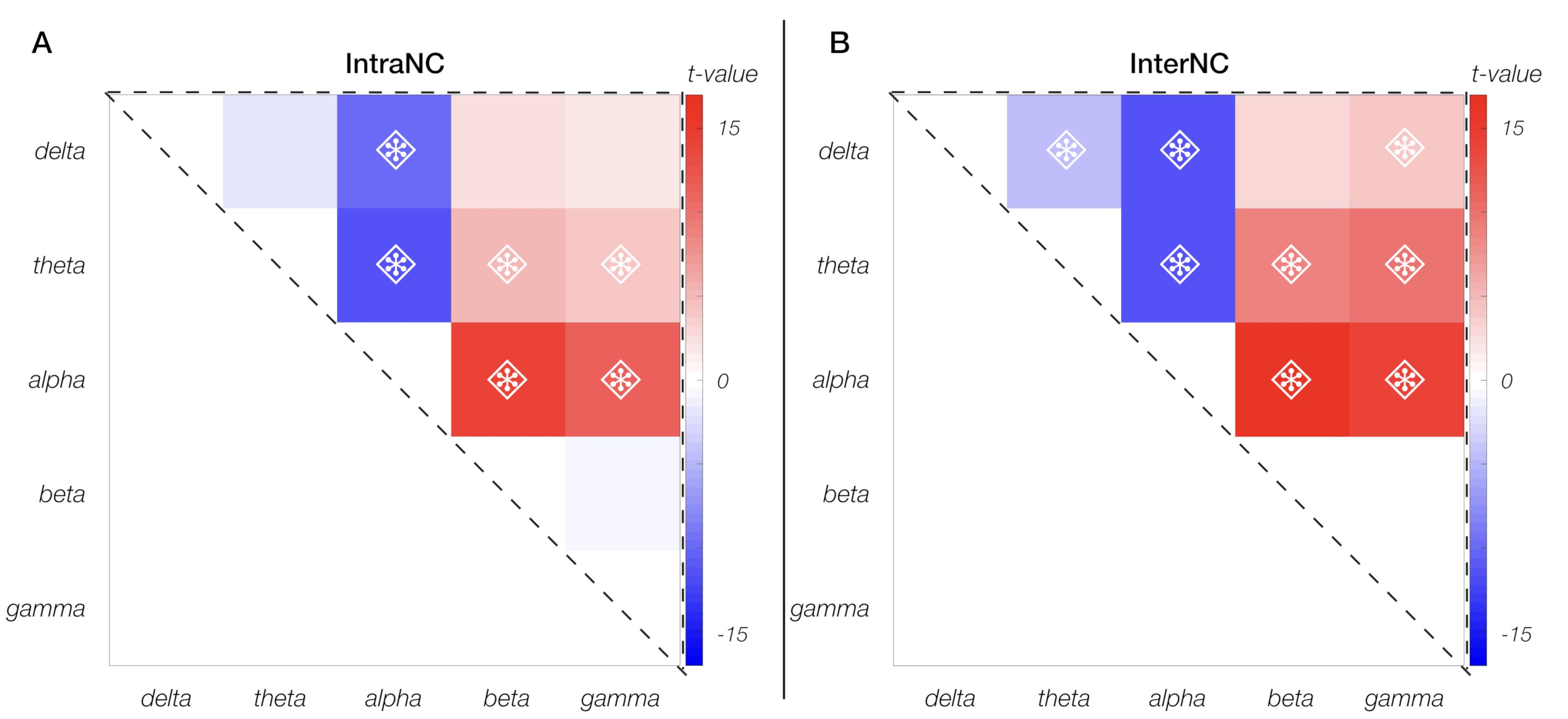

Supplement: Supplementary file 3 — Fig. S3. Differences in intra‐network (IntraNC) and inter‐network (InterNC) connectivity values between frequency bands. A two‐tailed paired t‐test was used to compare (A) IntraNC and (B) InterNC values, respectively, for each pair of frequency bands. Differences that are significant at p<0.001 are marked with an asterisk, and those at q<0.001 with a diamond. [file HBM-41-5187-s003.tiff]

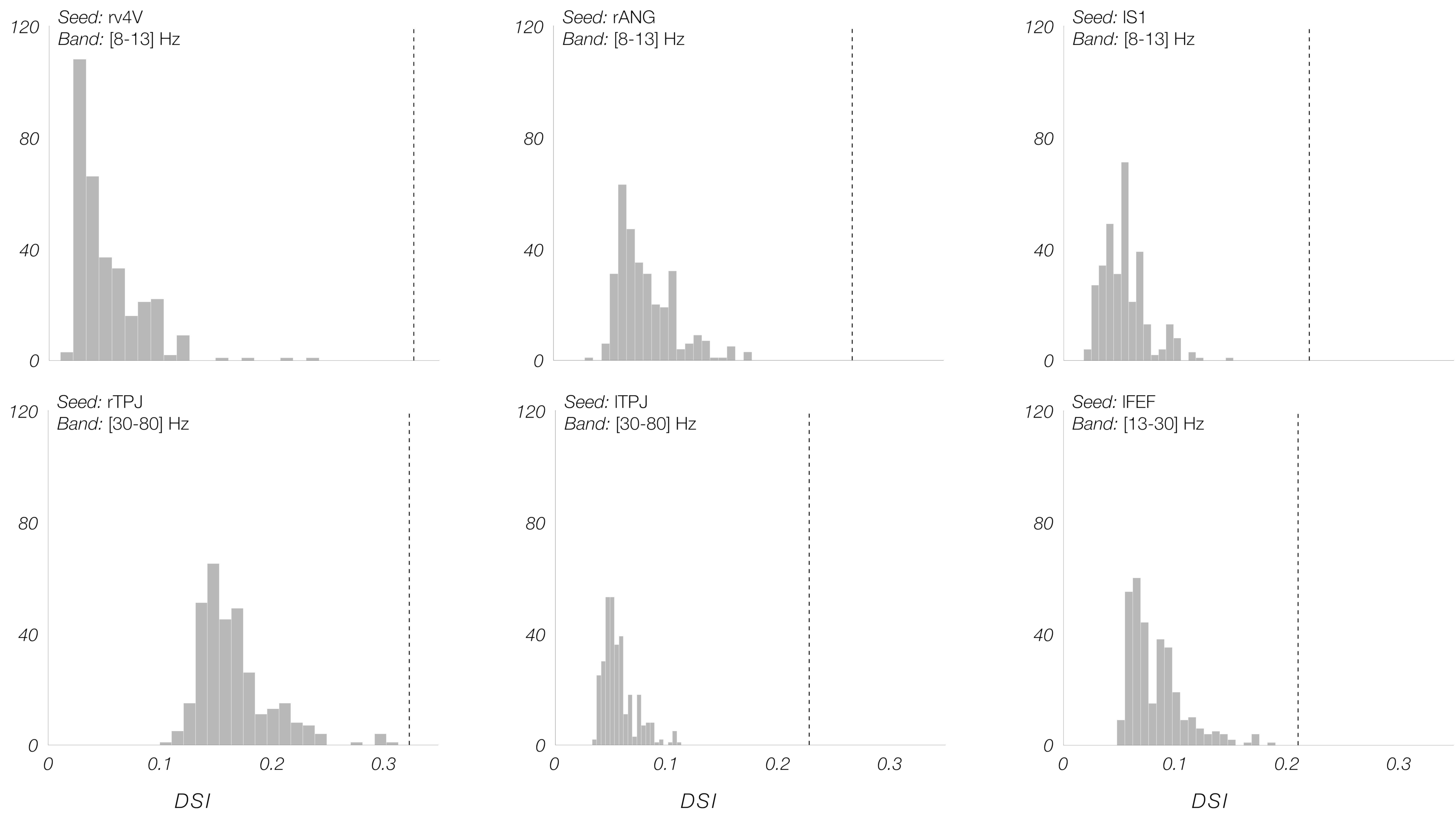

Supplement: Supplementary file 4 — Fig. S4. Dice similarity indices (DSI) between the EEG‐based and the fMRI‐based connectivity maps shown in Fig. 7 (clockwise from top left): rv4V (VN), rANG (DMN) and lS1 (SMN) in alpha band, rTPJ (VAN) and lTPJ (LAN) in gamma band, lFEF (DAN) in beta band. The actual DSI value are indicated in each panel with a dashed vertical line. A null‐distribution of DSI values, represented using a gray‐shaded histogram, was obtained by comparing the fMRI‐connectivity map with surrogate EEG‐connectivity maps. [file HBM-41-5187-s004.tiff]
