# Supplementary material for: Frequency‐dependent functional connectivity in resting state networks
Source: Hum Brain Mapp. 2020 Aug 25;41(18):5187–98. doi: 10.1002/hbm.25184 (PMC7670639; doi:10.1002/hbm.25184)
Supplement: Supplementary file 5 — Table S1 – For each network, the selected seeds and their coordinates in MNI space are indicated. [file HBM-41-5187-s005.pdf]

Table S1 – For each network, the selected seeds and their coordinates in MNI space are indicated.

Network name	*Seed name*	*Abbreviation*	*MNI coordinates*
Default Mode (DMN)	Posterior cingulate cortex	PCC	[5, -58, 29]
	Medial prefrontal cortex	MPFC	[-5, 35, -9]
	Left angular gyrus	lANG	[-57, -63, 17]
	Right angular gyrus	rANG	[56,-63,18]
Dorsal Attention (DAN)	Left Frontal Eye Field	lFEF	[-30,-9,52]
	Right Frontal Eye Field	rFEF	[30,-9,55]
	Left Inferior Parietal Sulcus	lIPS	[-27,-61,50]
	Right Inferior Parietal Sulcus	rIPS	[26,-60,48]
Ventral Attention (VAN)	Right Temporo-Parietal Junction	rTPJ	[60, -43, 16]
	Right Inferior Frontal Gyrus	rIFG	[42,5,1]
Language (LAN)	Left Temporo-Parietal Junction	lTPJ	[-54, -33, -4]
	Left Inferior Frontal Gyrus	lIFG	[-47, 14, 1]
Somatomotor (SMN)	Left Supplementary Motor Area	lSMA	[-1,-17,55]
	Left Primary Somatosensory Cortex	lS1	[-45,-17,49]
	Right Primary Somatosensory Cortex	rS1	[45,-17,49]
	Left Secondary Somatosensory Cortex	lS2	[-42,-13,10]
	Right Secondary Somatosensory Cortex	rS2	[42,-13,10]
Visual (VN)	Left human ventral Visual 4 area	lV4v	[-27,-81,-13]
	Right human ventral Visual 4 area	rV4v	[27,-81,-13]
	Left dorsal Visual 2 area	lV2d	[-45,-81,4]
	Right dorsal Visual 2 area	rV2d	[45,-81,4]
